# Evaluation of a worldwide EQA scheme for complex clonality analysis of clinical lymphoproliferative cases demonstrates a learning effect

**DOI:** 10.1007/s00428-021-03046-0

**Published:** 2021-03-08

**Authors:** Cleo Keppens, Elke Boone, Paula Gameiro, Véronique Tack, Elisabeth Moreau, Elizabeth Hodges, Paul Evans, Monika Brüggemann, Ian Carter, Dido Lenze, Maria Eugenia Sarasquete, Markus Möbs, Hongxiang Liu, Elisabeth M. C. Dequeker, Patricia J. T. A. Groenen

**Affiliations:** 1 Department of Public Health and Primary Care, Biomedical Quality Assurance Research Unit, University of Leuven, Kapucijnenvoer 35 block d, 1st floor, box 7001, 3000 Leuven, Belgium; 2 AZ Delta vzw - Laboratorium Moleculaire Diagnostiek, Deltalaan 1, 8800 Roeselare, Belgium; 3 Laboratory of Hemato-Oncology, Portuguese Institute of Oncology of Lisbon, Rua Prof Lima Basto, 1099-023 Lisboa, Portugal; 4 Precision Medicine Centre, Queen’s University Belfast, Health Science Building, 97 Lisburn Road, Belfast, BT9 7AE UK; 5 HMDS, Leeds Institute of Oncology, St. James University Hospital, Level 3 Bexley Wing Leeds, Leeds, LS9 7TF UK; 6 Department of Hematology, University Hospital Schleswig-Holstein, Langer Segen 8-10, 24105 Kiel, Germany; 7 Molecular Diagnostics, Histopathology, Nottingham University Hospitals NHS Trust, City Campus, Hucknall Rd., Nottingham, NG5 1PB UK; 8 Institut für Pathologie, Molekularpathologie, Charité –Universitätsmedizin Berlin, Chariteplatz 1, 10117 Berlin, Germany; 9 Laboratorio Biología Molecular, Servicio de Hematología, Hospital Universitario de Salamanca, Paseo de San Vicente, 58-182, 37007 Salamanca, Spain; 10 Molecular Malignancy Laboratory, Haematopathology and Oncology Diagnostic Service (HODS), Addenbrooke’s Hospital, Cambridge University Hospitals NHS Foundation Trust, Box 234, Hills Road, Cambridge, CB2 0QQ UK; 11 Department of Pathology, Radboud University Medical Centre Nijmegen, Geert Grooteplein Zuid 10, 6525 GA Nijmegen, The Netherlands

**Keywords:** External quality assessment, Clonality analysis, IG rearrangements, TCR rearrangements

## Abstract

Clonality analysis of immunoglobulin (IG) or T-cell receptor (TR) gene rearrangements is routine practice to assist diagnosis of lymphoid malignancies. Participation in external quality assessment (EQA) aids laboratories in identifying systematic shortcomings. The aim of this study was to evaluate laboratories’ improvement in IG/TR analysis and interpretation during five EQA rounds between 2014 and 2018. Each year, participants received a total of five cases for IG and five cases for TR testing. Paper-based cases were included for analysis of the final molecular conclusion that should be interpreted based on the integration of the individual PCR results. Wet cases were distributed for analysis of their routine protocol as well as evaluation of the final molecular conclusion. In total, 94.9% (506/533) of wet tests and 97.9% (829/847) of paper tests were correctly analyzed for IG, and 96.8% (507/524) wet tests and 93.2% (765/821) paper tests were correctly analyzed for TR. Analysis scores significantly improved when laboratories participated to more EQA rounds (*p*=0.001). Overall performance was significantly lower (*p*=0.008) for non-EuroClonality laboratories (95% for IG and 93% for TR) compared to EuroClonality laboratories (99% for IG and 97% for TR). The difference was not related to the EQA scheme year, anatomic origin of the sample, or final clinical diagnosis. This evaluation showed that repeated EQA participation helps to reduce performance differences between laboratories (EuroClonality versus non-EuroClonality) and between sample types (paper versus wet). The difficulties in interpreting oligoclonal cases highlighted the need for continued education by meetings and EQA schemes.

**Supplementary Information:**

The online version contains supplementary material available at 10.1007/s00428-021-03046-0.

## Introduction

Clonality testing is widely accepted as a valuable tool in routine diagnosis of lymphoid malignancies [1, 2]. The vast majority of lymphoid malignancies arise from the unconstrained expansion of a single transformed B- or T-cell, accompanied by the presence of clonal rearrangements of immunoglobulin (IG) or T-cell receptor (TR) genes, rendering them the most widely applied gene targets for clonality testing.

In 2003, the European BIOMED-2 consortium, continued under the name “EuroClonality consortium,” designed a standardized multiplex PCR assay for nearly all IG/TR targets [3], which showed a high rate of clonality detection in common B- and T-cell malignancies [4–12]. To date, several commercial kits are available to run the multiplex PCR assays.

Due to the technical standardization and commercialization (PCRs, protocols, and readouts), clonality assays can be performed in routine diagnostics [13]. However, reporting of clonality assays is still considered a complex task, because molecular clonality testing reflects immunobiology and comprises the integrated interpretation of multiple multiplex PCR results. These multiplex PCRs use primers of potentially different efficiencies, annealing to highly homologous genes. Although there are basic rules for interpretation of the molecular patterns [13], extensive knowledge of IG and TR gene rearrangement patterns and the PCR design is needed. Also, interpretation should consider the pathology and the clinical question as the presence of a small clone in a reactive lesion has another implication than its presence in a full-blown malignancy.

Laboratories performing molecular pathology tests are advised to participate in external quality assessment (EQA) schemes [14], preferably an accredited scheme providing samples mimicking routine cases as closely as possible [15]. It is essential that the EQA participants read the final reports with feedback on errors made by all participants, act on recommendations made, and ensure that their own errors are corrected rapidly [14].

The EuroClonality consortium organized five EQA rounds between 2008 and 2011 [16], using capillary electrophoresis (GeneScan, GS) or polyacrylamide heteroduplex (HD) gel analysis [3]. The schemes aimed to (i) assess the laboratory performance and (ii) develop a uniform scoring system for interpretation of IG/TR clonality testing. To render interpretation less subjective, algorithms have been introduced, especially in the USA [17–19]. However, this may potentially lead to false negative or false positive interpretation [13], and the need for guidelines on interpretation and reporting of clonality data is apparent for IG/TR routine diagnostics and EQA schemes. This prompted the development of the EuroClonality (BIOMED-2) guidelines. The guidelines describe the technical scoring of the individual IG and TR PCR target results, and scoring of the final molecular conclusion, based on the integration of the different PCR results. During validation of the EuroClonality uniform description and reporting system, the majority of the cases were scored appropriately, with only 3.1% of 1150 cases being identified as difficult to score, i.e., the final scoring of either a minor clone with polyclonal background or polyclonal with a minor background, actually describe the same phenomenon but the scoring may reflect the personal favor of the clinical scientist [13].

Several other providers offer IG/TR EQA schemes [20–24]. Both small sample sets with frequent distribution and larger sample sets are currently used in different EQA programs [25, 26] (**supplemental Table** 1). Previously, performance improvement upon EQA participation or other quality improvement projects has been reported in schemes for testing of oncological biomarkers [27, 28], but not yet for clonality testing.

The aim of this paper was to investigate the effect of repeated EQA participation on the laboratories’ performance for complex clonality analysis. Important parameters such as the participant group, the different final molecular interpretations (clonal, polyclonal, oligoclonal; without evaluation of the more detailed molecular interpretation), sample information, and the analysis method were integrated in these analyses. The data are based on the results of five EQA rounds for IG and TR rearrangement analysis in suspected lymphoproliferations between 2014 and 2018.

## Materials and methods

### EQA scheme set-up

**Fig. 1:**
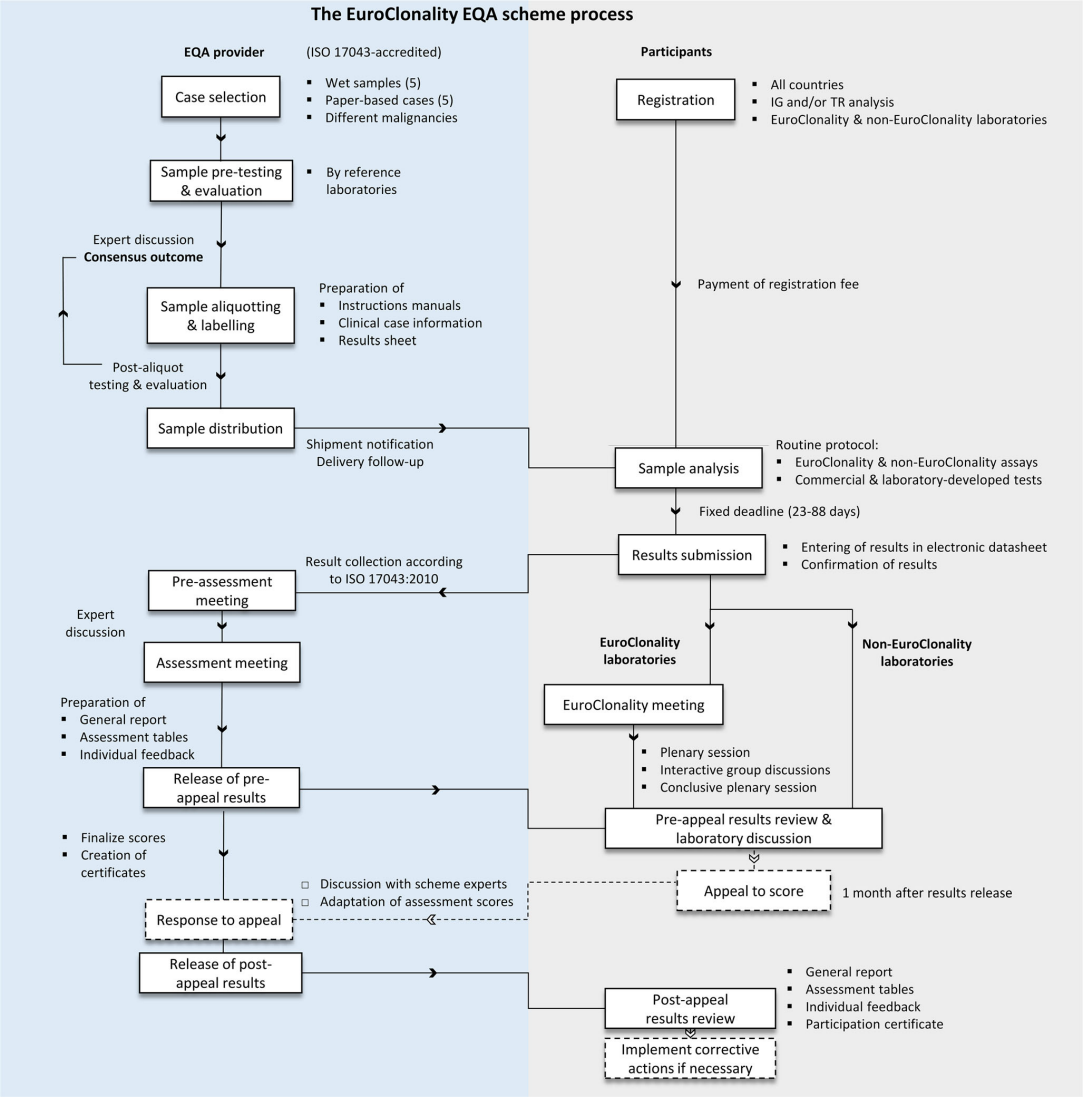
Overview of the EuroClonality EQA scheme process. EQA, external quality assessment; IG, immunoglobulin gene; TR, T-cell receptor gene

The schemes were organized by the EuroClonality consortium [29] in collaboration with the Biomedical Quality Assurance Research Unit of KU Leuven as the coordination center, accredited conforming to ISO/IEC 17043:2010 [30]. Each EQA round comprised analysis of extracted DNA samples and interpretation of clonality patterns from paper-based cases on a total of 10 clinical cases. In addition to the EuroClonality laboratories who were involved in the development of EuroClonality/BIOMED-2 primer sets and protocols [3–5] and are members of the EuroClonality consortium, also non-EuroClonality laboratories could register. Enrolled laboratories could opt to participate in IG or TR testing, or both. The EQA scheme process is depicted in Fig. 1.

### Sample selection

Participants received five cases for IG clonality testing and/or five cases for TR clonality testing. With the exception of 2014 (only paper-based cases), these cases alternated yearly to include three DNA samples and two paper-based cases in a given year, versus two DNA and three paper cases in the next year (Table 1). As both hemato-oncology and pathology labs perform clonality analysis, cases of different sample types (e.g., peripheral blood, fresh tissue, FFPE tissue) were included, reflecting the clinical diagnostics. The selection for the wet cases was based on the availability of samples with sufficient DNA-yield for testing in an EQA, the representation of the tube patterns, and results from previous EQA rounds. The selection for the paper-based cases was based on the representation of the tube patterns, the evaluation of rearrangement patterns of separate tubes into an integrated conclusion, and the results from previous EQA rounds. Only wet and paper cases with a consensus overall molecular interpretation during pretesting were included.

**Table 1:** Performance parameters for IG/TR clonality testing over time of the EQA participants combined

EQA scheme year	Determination of correct result***	Deadline for results submission (days)	Wet cases distributed	Paper-based cases provided	# registered participants	# participants who returned results	# countries	Average score on 5 points**	% participants with max score	% successful participants (score ≥4/5)*	% successful participants in 2 most recent schemes (score ≥9/10)* (*N)*	% correct results wet cases (*N*)	% correct results paper cases (*N)*	% total correct, results (*N*)
IG rearrangement testing														
2014	Consensus	23	0	5	63	62	15	4.90	90.3	100.0	/	/	98.1 (310)	98.1 (310)
2015		30	3	2	58	57	13	4.72	84.2	92.9	94.1 (51)	93.6 (171)	95.6 (114)	95.6 (285)
2016	Expert panel	39	2	3	55	54	13	4.75	75.9	96.3	95.1 (41)	88.9 (108)	98.1 (162)	94.4 (270)
2017		69	3	2	48	48	12	4.98	97.9	100.0	97.4 (38)	99.3 (144)	100.0 (96)	99.6 (240)
2018		88	2	3	55	55	15	4.87	89.1	98.2	100.0 (40)	97.3 (110)	97.6 (165)	97.5 (275)
TR rearrangement testing														
2014	Consensus	23	0	5	61	60	15	4.87	88.3	98.3	/	/	97.3 (300)	97.3 (300)
2015		30	2	3	57	56	13	4.82	82.1	100.0	97.9 (49)	93.8 (112)	98.2 (168)	96.4 (280)
2016	Expert panel	39	3	2	52	51	13	4.55	33.3	90.2	89.7 (39)	96.7 (153)	66.7 (102)	84.7 (255)
2017		69	2	3	47	47	12	4.78	80.9	97.9	77.8 (36)	98.9 (94)	93.6 (141)	95.7 (235)
2018		88	3	2	55	55	15	4.89	89.1	100.0	92.5 (40)	97.6 (165)	98.2 (110)	97.8 (275)

*EQA*, external quality assessment; *IG*, immunoglobulin gene; *N*, number; *TR*, T-cell receptor gene

^*^Successful participation is defined as a score of ≥4/5. Successful participation after two participations is defined as a score of ≥ 9/10 for both schemes combined. The number of participants does not necessarily equal the number of participants who submitted results, given that not all laboratories participated during the previous EQA scheme and the performance over 2 EQA rounds was not calculated

^**^One paper case from 2015 was considered an educational sample to calculate the TR performance score; all participants received 1 point as no consensus outcome could be reached

^***^In 2016, 0.5 points were deducted in case there was a discrepancy between individual tubes and final conclusion or wrong identification of clonal peak for one specific case

Wet samples consisted of color-coded tubes containing 40 μL DNA at a concentration of 25–50 ng/μL. Paper-based cases focused on the interpretation of IG/TR GS patterns, created by duplicate fragment analysis (GS) of PCR products on various Genetic Analyzer Systems (Life Technologies, Beckman Coulter). Quality of the DNA samples was assessed with the EuroClonality/BIOMED-2 quality control-gene PCR (100, 200, 300, 400 bp amplicons), and the largest sized amplicon product detectable was reported. Participants received information on the PCR targets (e.g., FR1-JH for IGH tube A), fluorochromes (FAM or HEX), and size standards (e.g., LIZ500, ILS600). Patterns were provided per BIOMED-2 tube, including a full view of the tube patterns and a zoomed view per sample to aid visualization of the case’s overall GS profile. All samples (paper and wet) were presented as clinical cases and relevant clinical details (sample type, age/sex of patient, suspected diagnosis, and request), and flow cytometry, histomorphology, and/or immunostaining data were provided.

### Results of clonality analysis: the individual tests as well as the final molecular interpretation

Participants were asked to analyze all cases using their routine protocols and to interpret the results according to the published guidelines [13]. Results were entered in an electronic datasheet (Formdesk) and included (i) the overall molecular interpretation, (ii) an optional more detailed interpretation, and (iii) a technical description per PCR tube (with or without peak size(s)). Additionally, information about the detection technique (HD or GS) and test assay used (only in 2018) were requested.

### Evaluation and feedback

In 2014 and 2015, a consensus (overall) molecular interpretation and result per PCR tube was reached based on the concerted discussion of the participants’ data. From 2016 up to 2018, consensus scoring was established by the EQA committee experts for the wet and paper cases prior to distribution.

In all scheme years, a maximum score of 1 point could be obtained per sample for a correct final molecular interpretation (Table 1). As the results of the different multiplex PCRs were used to form the basis for the final molecular interpretation, the individual PCR tube results were not scored. For particular cases, a more detailed interpretation of the final molecular interpretation was required (out of scope for this paper). Only in 2016 and 2017, half a point was additionally deducted for an incorrect or suboptimal detailed interpretation. In 2016, half a point was also deducted for discrepancies between individual tubes and the final conclusion or an incorrect identification of clonal peaks.

In the yearly EuroClonality meeting, the results of the EQA scheme were discussed, starting with a plenary presentation of the results, followed by detailed small group discussions involving the expert EQA committee members and the EuroClonality consortium participants. Finally, there was a summarizing plenary presentation. Analysis of the EQA data was integrated and described in detail in an educational EQA report and provided to all scheme participants. The general scheme summary included detailed information about the molecular conclusion and per tube PCR results, an assessment table with scores per case, and a participation certificate [25]. The criterion for successful participation was a performance rate of ≥80% in that respective scheme year, corresponding to at least 4 out of 5 correct final molecular interpretations. Laboratories with a score of ≤4.5 on 5 received a warning due to possible risk of unsatisfactory performance after two EQA rounds. Laboratories with a score of at least 90% (9/10) [25] in two subsequent EQA rounds were listed on the EuroClonality website [29, 31].

### Statistics

Statistics were performed with IBM SPSS Statistics v25 (IBM, Armonk, NY, USA) with significance levels set at *α*=0.05. Mann-Whitney *U* (MWU) tests were performed to evaluate differences in average analysis scores between groups, and Kruskal-Wallis (KW) tests to assess improvement upon repeated participation for a given group.

## Results

### General overview

Over all schemes between 2014 and 2018, 84 unique laboratories from 17 countries participated, of which 27 (32.1%) were EuroClonality members, resulting in 279 and 272 scheme registrations for IG and TR analysis, respectively. Results were returned for 98.9% (276/279) and 98.9% (269/272) IG and TR participations, respectively (Table 1).

Average scores were high with minimum values of 4.72/5 (2015, IG) and 4.55/5 (2016, TR) (Table 1). The percentage of laboratories obtaining the maximum score (5/5 points) varied between 75.9–97.9% for IG and 33.3–89.1% for TR analysis.

For IG and TR, a total of 10 wet cases and 15 paper-based cases were distributed to multiple registered laboratories (Table 1). In summary, 94.9% (506/533) of wet and 97.9% (829/847) of paper tests were correct for IG (MWU, *p*=0.144), versus 96.8% (507/524) wet and 93.2% (765/821) paper tests for TR (MWU, *p*=0.686). For all sample types and diagnoses, 87.5% or more of the participants were able to provide the correct consensus outcome (Table 2). This excludes a paper-based case in 2016 (peripheral blood with relapsed T-cell prolymphocytic leukemia) for which 29.4% (15/51) of participants incorrectly interpreted this difficult case with oligoclonality/multiple clones detected (**Supplemental Table** 2).

**Table 2:** Performance related to sample origin and clinical diagnosis for the different cases distributed in the EQA schemes

Sample type/final clinical diagnosis	*N* tests	% correct results	IG		TR	
			Paper	Wet	Paper	Wet
Bone marrow	434	99.4	99.6	100.0	98.4	100.0
Chronic lymphocytic leukemia	54	100.0	100.0			
Hairy cell leukemia	62	98.4	98.4			
Large granular lymphocyte leukemia	47	100.0			100.0	
Monoclonal B-cell lymphocytosis	48	100.0	100.0			
Multiple myeloma, relapse	54	100.0		100.0		
Mycosis fungoides (with extra-cutaneous dissemination)	60	96.7			96.7	
Plasmablastic lymphoma	62	100.0	100.0			
Reactive lesion	47	100.0				100.0
Lachrymal node FFPE, reactive lesion	54	96.3	96.3			
Lymph node FFPE	219	95.0		91.2	98.2	92.2
Follicular lymphoma (with t(14;18) detected by FISH)	57	91.2		91.2		
Peripheral T-cell lymphoma, NOS	56	100.0			100.0	
Reactive lesion	51	92.2				92.2
T-cell prolymphocytic leukemia	55	96.4			96.4	
Lymph node fresh	532	95.4		94.8		96.4
Angioimmunoblastic T-cell lymphoma	55	100.0				100.0
Extraosseous plasmacytoma	55	100.0		100.0		
Follicular lymphoma (with t(14;18) detected by FISH)	102	90.8		90.8		
Mature B-cell neoplasm	57	94.7		94.7		
Peripheral T-cell lymphoma, NOS	102	99.0				99.0
Reactive lesion	161	93.4		96.3		87.5
Paravertebral mass FFPE, diffuse large B-cell lymphoma, NOS	48	100.0	100.0			
Peripheral blood	449	89.5	98.4		73.6	99.4
Mature B-cell neoplasm	62	100.0	100.0			
Peripheral T-cell non-Hodgkin lymphoma, NOS	51	100.0				100.0
Reactive lymphocytosis	118	95.7	96.8		94.6	
Relapsed T-cell acute lymphoblastic leukemia	51	98.1				98.1
Relapsed T-cell prolymphocytic leukemia	51	29.4			29.4	
Sézary syndrome	60	96.7			96.7	
T-cell large granular lymphocytic leukemia	56	100.0				100.0
Pleural fluid, peripheral T-cell lymphoma, NOS	55	100.0			100.0	
Skin tissue FFPE	102	95.5			100.0	90.9
Diagnosis not conclusive (early phase of parapsoriasis or mycosis fungoides could not be excluded) and further clinicopathological follow-up was advised	47	100.0			100.0	
Reactive lesion	55	90.9				90.9
Skin tissue fresh	394	95.4	97.6		94.5	
Extranodal marginal zone B-cell lymphoma	54	100.0	100.0			
Mycosis fungoides	218	93.9			93.9	
Peripheral T-cell lymphoma, NOS	60	96.7			96.7	
Primary cutaneous follicle left lymphoma	62	95.2	95.2			
Spleen tissue FFPE, diffuse large B-cell lymphoma, NOS	112	100.0	100.0			
Spleen tissue fresh, nodal marginal zone lymphoma (with transformation to large B-cell lymphoma)	48	100.0		100.0		
Stomach tissue fresh, chronic gastritis	57	91.2	91.2			
Thyroid gland isthmus FFPE, reactive lesion	55	94.5		94.5		
Tumor tissue FFPE, plasma cell neoplasm	55	92.7	92.7			
Umbilicus fresh, diffuse large B-cell lymphoma, NOS	55	100.0	100.0			

Data excludes one spleen tissue FFPE case (2015, paper TR case) with final clinical diagnosis of diffuse large B-cell lymphoma for which no consensus could be reached

*EQA*, external quality assessment; *FFPE*, formalin-fixed paraffin embedded; *FISH*, fluorescence in situ hybridization; *IG*, immunoglobulin gene; *N/A*, not applicable; *NOS*, not otherwise specified; *TR*, T-cell receptor gene

### Improvement related to repeated EQA participation

**Fig. 2:**
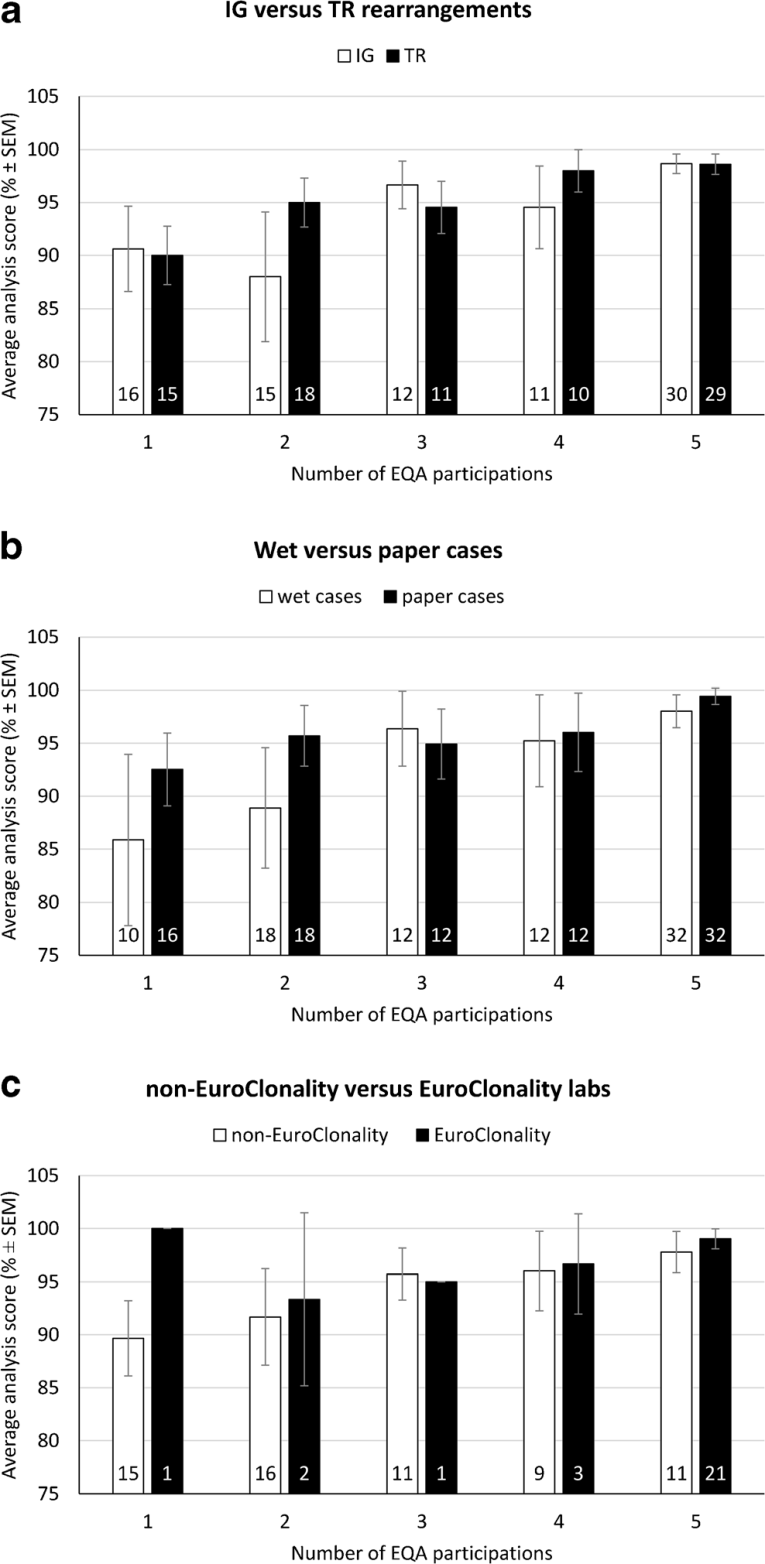
Improvement upon repeated EQA participation for the different targets, sample, and participant types. #, number; IG, immunoglobulin gene; SEM, standard error of the mean; TR, T-cell receptor gene. Bar numbers represent the number of unique laboratories for which the average analysis score is calculated. No standard errors are shown for bars with only one participant

We evaluated the performance on individual laboratory level based on the number of EQA participations, not related to the general average score for that scheme year. The average analysis scores were significantly higher for individual laboratories who participated in multiple EQA scheme rounds (KW, *p*=0.001) for IG and TR. In fact, the successful IG testing performance was 90.6% for first time participants (*n*=16 laboratories), versus 98.7% for laboratories who participated five times (*n*=30) (Fig. 2, panel A) (KW, *p*=0.133). For TR analysis, laboratories who participated one time reached an average score of 90.0% (*n*=15), whereas 5th time participants reached on average 98.6% (*n*=29) (KW, *p*=0.011).

Overall, laboratories performed better for the paper-based cases as compared to the wet cases, although not significant (MWU, *p*=0.466) (Fig. 2, panel B). During a first EQA participation, a score of 85.9% was reached for wet cases, compared to 92.5% for paper-based cases (IG and TR combined). The difference between both sample types decreased upon frequent EQA participation, ultimately reaching scores of 98.0% (wet cases) and 99.4% (paper cases).

Both EuroClonality and non-EuroClonality laboratories benefited from repeated EQA participation. The EuroClonality laboratories performed significantly better compared to non-EuroClonality participants (MWU, *p*=0.008) (Fig. 2, panel C). Better performance by EuroClonality laboratories was also observed for IG and TR testing for paper and wet cases separately, although only significant for the paper cases (MWU, *p*=0.007 for paper cases, *p*=0.149 for wet cases, *p*=0.057 for IG, *p*=0.068 for TR) (**Supplemental Figure** 1)**.**

### Evaluation of the different final molecular interpretations

In total, 1380 tests (both on wet and paper-based cases) were performed for IG rearrangements (Table 3). A total of 97.5% (1021/1047) and 96.1% (265/276) tests correctly assigned the final interpretation of clonal or polyclonal, respectively. Note that the more detailed molecular interpretations were evaluated but no points were deduced when the more detailed interpretation was not correct. Only 12.3% (7/57) of tests with a consensus outcome of oligoclonality/multiple clones were correct, as 78.9% (45/57) were reported as clonal. For TR analysis, 1345 tests were performed (Table 3), of which one paper-based case in 2015 was considered to be educational since no consensus outcome was reached. Similar to IG, the majority of clonal (893/913, 97.8%) and polyclonal (251/269, 93.3%) TR tests were correct, while the majority of oligoclonal tests were incorrectly assigned as clonality detected (76/107, 71.1%). Incorrect interpretations were more often observed for wet samples compared to paper cases, especially for IG analysis (except for oligoclonality, which only included paper-based cases).

**Table 3:** Percentage of correct and incorrect final molecular interpretations in wet and paper-based cases

Final molecular interpretation in EQA schemes 2014–2018	IG analysis			TR analysis		
	*N*=1380	Paper	Wet	*N*=1345	Paper	Wet
Consensus: clonality detected	1047 (75.9)	674 (64.4)	373 (35.6)	913 (67.9)	551 (60.4)	362 (39.6)
Clonality detected	1021 (97.5)	666 (65.2)	355 (34.8)	893 (97.8)	533 (59.7)	360 (40.3)
Polyclonality detected (no clonality detected)	19 (1.8)	6 (31.6)	13 (68.4)	9 (1.0)	8 (88.9)	1 (11.1)
No rearrangement in IG/TR targets detected	5 (0.5)	1 (20.0)	4 (80.0)	1 (0.1)	1 (100.0)	0 (0.0)
No clonality detected, suggestive of low template amount	1 (0.1)	0 (0.0)	1 (100.0)	0 (0.0)	/	/
Oligoclonality/multiple clones detected	1 (0.1)	1 (100.0)	0 (0.0)	7 (0.8)	6 (85.7)	1 (14.3)
Not evaluable	0 (0.0)	/	/	3 (0.3)	3 (100.0)	0 (0.0)
Consensus: oligoclonality/multiple clones detected	57 (4.1)	57 (100.0)	0 (0.0)	107 (7.9)	107 (100.0)	0 (0.0)
Oligoclonality/multiple clones detected	7 (12.3)	7 (100.0)	0 (0.0)	27 (25.2)	27 (100.0)	0 (0.0)
Clonality detected	45 (78.9)	45 (100.0)	0 (0.0)	76 (71.1)	76 (100.0)	0 (0.0)
Polyclonality detected (no clonality detected)	5 (8.8)	5 (100.0)	0 (0.0)	3 (2.8)	3 (100.0)	0 (0.0)
Not evaluable	0 (0.0)	/	/	1 (0.9)	1 (100.0)	0 (0.0)
Consensus: polyclonality detected (no clonality detected)	276 (20.0)	116 (42.0)	160 (68.0)	269 (20.0)	107 (39.8)	162 (60.2)
Polyclonality detected (no clonality detected)	265 (96.1)	112 (42.3)	153 (57.7)	251 (93.3)	105 (41.8)	146 (58.1)
No rearrangement in IG/TR targets detected	7 (2.5)	4 (57.1)	3 (42.9)	5 (1.9)	0 (0.0)	5 (100.0)
Clonality detected	2 (0.7)	0 (0.0)	2 (100.0)	13 (4.8)	2 (15.4)	11 (84.6)
No clonality detected, suggestive of low template amount	2 (0.7)	0 (0.0)	2 (100.0)	0 (0.0)	/	/
No consensus: educational case	0 (0.0)	/	/	56 (4.2)	56 (100.0)	0 (0.0)
Clonality detected	0 (0.0)	/	/	31 (55.4)	31 (100.0)	0 (0.0)
Polyclonality detected (no clonality detected)	0 (0.0)	/	/	22 (39.3)	22 (100.0)	0 (0.0)
No clonality detected, suggestive of low template amount	0 (0.0)	/	/	2 (3.6)	2 (100.0)	0 (0.0)
Not evaluable	0 (0.0)	/	/	1 (1.8)	1 (100.0)	0 (0.0)

*EQA*, external quality assessment; *IG*, immunoglobulin gene; *N*, number of samples tested; *TR*, T-cell receptor gene

### Evaluation of the analysis methods used by the EQA participants

For IG and TR analysis of the wet samples, 90.8% (*n*=533) and 92.2% (*n*=524) of tests were analyzed by GS. The remaining tests were analyzed by HD (8.3% for IG and 7.3% for TR), which is also described as a preferred analytical technique for some multiplex PCR-tubes [3]. For IG analysis, the majority of the participants tested the IGH-A (94.6%), IGH-B (98.7%), and IGH-C (98.9%) tubes (**Supplemental Table** 3). These three tubes were mainly tested by EuroClonality Invivoscribe reagents (30/55 participants), EuroClonality laboratory-developed tests (LDT) (23/55), non-EuroClonality LDT (1/55), and Invivoscribe next-generation sequencing (NGS) reagents (1/55).

For wet TR testing, the most included tubes were TRG-A and TRG-B tubes (97.5%). For these two tubes, EuroClonality Invivoscribe reagents were mainly used (29/55 participants), followed by EuroClonality LDT (22/55), and non-EuroConality LDT (2/55). One participant used Invivoscribe NGS reagents, and one other laboratory did not test these targets. Reagents for the other tubes are shown in **Supplemental Table** 3.

## Discussion

The BIOMED-2/EuroClonality assays are widely used for clonality testing of suspected lymphoproliferations. Clonality testing is not a stand-alone test but is an important integral part in the diagnosis of lymphoid malignancies. Correct analysis, evaluation, and result reporting are indispensable and contribute to a correct diagnosis. Particularly, the appropriate interpretation of clonality assays requires study, learning, and training on the job. This can be facilitated by participation in EuroClonality educational workshops, or by submitting difficult cases via the EuroClonality website to get online support. In this paper, we show that participation in the EuroClonality EQA schemes significantly contributes to improving the diagnostic interpretation.

The overall performance scores for both IG and TR analysis were high, with more than 90% of successful participants each year. The individual participants had a significantly higher score when participating in more EQA rounds, although there was no obvious overall improvement between 2014 and 2018. There was no observed difference in performance based on the sample type or final clinical diagnosis. The used methodology could not be linked to the improvement for a specific laboratory or sample type.

The final molecular interpretations such as “clonal” or “polyclonal” were in general scored well. Also, truly challenging cases were included such as clonal cases with polyclonal background, cases with bi-allelic rearrangements, bi-clonal cases, and cases with multiple clonal (IGK or TRB) rearrangements that still belong to one clone. In our schemes, thus far, we have evaluated the more detailed molecular interpretations such as bi-allelic or bi-clonal, but no points were deducted when the more detailed interpretation was incorrect. In the next EQA schemes, we intend to include the more detailed interpretations in the evaluation. Based on the previous EQA schemes, we then expect a lower performance for clonal samples. The scoring of oligoclonality clearly was difficult. Oligoclonality is defined as the reproducible detection of three or more clones. As for the interpretation of clonality (including bi-allelic or bi-clonal cases), this requires the appropriate interpretation of the individual tube results as well as understanding the IG and TR loci and PCR design. Due to the non-quantitative PCR nature and potentially preferential amplification of some rearrangements, the identification of true clonal rearrangements compared to minor peaks in an irregular polyclonal background is especially difficult for oligoclonal cases. Because true oligoclonal cases are scarce, the experience with these cases is limited. Only three oligoclonal cases could be included in the paper-based EQA; one for IG and TR in 2015 and one TR case in 2016. Of the total 164 molecular interpretations (Table 3), EuroClonality laboratories correctly assigned the cases as oligoclonal in 24/73 (32.9%) tests, compared to 10/91 (11.0%) for non-EuroClonality laboratories. Of the 56 laboratories interpreting the first oligoclonal TR case in 2015, 39 participated again in 2016. Twenty-eight of 39 participants incorrectly denoted the oligoclonal case in 2015, of which 21 made the same mistake and 7 laboratories improved in 2016. The other 7 participants had a correct outcome in both 2015 and 2016, while 4 laboratories were correct in 2015, but incorrect in 2016.

A better performance (although not significant) was observed for paper-based tests in which only the result interpretation was evaluated, versus the wet cases in which the technological approach, performance of the test, and the interpretation of the results were evaluated. While paper cases were evaluated adequately during a laboratory’s first or second EQA participation, wet cases were more error-prone (Fig. 2, panel B). This is not surprising, given that wet sample analysis includes extra (pre-)analytical processes potentially impacting the results, compared to solely interpreting GS results according to guidelines. However, paper-based cases may also be perceived as difficult by the laboratories, as cases with complex rearrangement patterns were included.

Both EuroClonality and non-EuroClonality laboratories improved their performance upon repeated participation, which is in line with results for biomarker analysis in colorectal cancer [28]. The overall scores were significantly better for EuroClonality laboratories (Fig. 2, panel C). The EuroClonality-affiliated participants may have benefited from the annual meeting and the provided feedback in the group discussions. Feedback has been shown to be an important parameter in learning [32]. However, the better performance should be interpreted with caution, as the majority of the EuroClonality-affiliated laboratories also participated more frequently to the EQA schemes, and repeated participation significantly improved performances. In addition, the difference between EuroClonality and non-EuroClonality laboratories is smaller for individual scheme years. We expected a larger score difference in a single scheme, given the longstanding experience of the EuroClonality participants, who were involved in the design and testing phase of the BIOMED-2/EuroClonality assays and in preparation of the EuroClonality guidelines. The question remains how non-EuroClonality laboratories educated themselves. Most likely, training on the job within an expert environment and/or attending dedicated trainings resulted in translation of theoretical knowledge into diagnostic practice and competence building. In addition, the feedback given in the extended EQA report may also have contributed to learning and good performance [33, 34]. In the end, it remains the responsibility of the participants to implement the necessary corrective actions to improve performance.

This EQA scheme with five wet cases and five paper-based cases in each round allowed us to evaluate the successful performance over two EQA rounds, as it was previously estimated that at least 10 samples are needed to allow a reliable performance estimate [25, 26]. Accredited laboratories have to demonstrate their performance, but not all laboratories participated to all EQA rounds, as the frequency of participation is not specified by ISO15189 [15] or equivalent national accreditation standards. Recent recommendations from the Belgian Molecular Diagnostics working group now state that laboratories should perform a risk analysis to determine their ideal participation frequency [35].

The several international clonality EQA providers each (i) evaluate a different number of IG/ TR targets, (ii) distribute various numbers of samples per annum, and (iii) apply different criteria for successful participation (**Supplemental Table** 1). Although not all providers include paper-based cases, the majority assesses the laboratory’s interpretation of the rearrangement patterns according to the guidelines. Namely, EQA providers should assess the complete analysis process, from pre-analytical to post-analytical phase [25]. As the participants received pre-extracted DNA samples, the DNA extraction and preparation steps are not evaluated in the EQA scheme, and could impose additional difficulties in a routine setting. The cut-off of 80% for successful performance and 90% after two participations in these EQAs was based on the requirements for EQA programs, which recommend a cut-off of 90% assessed on a total of 10 samples [25]. Similar to the harmonization efforts in molecular oncology, increased harmonization between providers is advisable for clonality analysis to define a uniform scope, scoring system, criteria for successful participation, and actions following unsatisfactory performance in Europe [36].

In summary, we observed a high performance for IG and TR analysis, which increased when participating to more EQA rounds. There was a higher performance for paper-based cases compared to wet cases and for EuroClonality compared to non-EuroClonality laboratories. There was no difference related to the EQA scheme year, sample origin, or clinical diagnosis. The observed difficulties in interpreting oligoclonal cases highlight the need for continued education via meetings and EQA schemes.

## Supplementary Information

ESM 1 (PDF 227 kb).

ESM 2 (PDF 407 kb).

ESM 3 (PDF 341 kb).

ESM 4 (PDF 345 kb).

## Data Availability

The datasets generated during and/or analyzed during the current study are available from the corresponding author on reasonable request.

## Abbreviations

EQA: External quality assessment
GS: GeneScan
HD: Heteroduplex
IG: Immunoglobulin gene
KW: Kruskal-Wallis test
LDT: Laboratory-developed test
MWU: Mann-Whitney *U* test
NGS: Next-generation sequencing
PCR: Polymerase chain reaction
TR: T-cell receptor gene

## References

[1] Van Dongen JJM, Wolvers-Tettero IL (1991). Analysis of immunoglobulin and T cell receptor genes, part II. Clin Chim Acta.

[2] Van Krieken JHJM, Langerak AW, Macintyre EA (2007). Improved reliability of lymphoma diagnostics via PCR-based clonality testing. Report of the BIOMED-2 Concerted Action BHM4-CT98-3936. Leukemia.

[3] Van Dongen JJ, Langerak AW, Bruggemann M (2003). Design and standardization of PCR primers and protocols for detection of clonal immunoglobulin and T-cell receptor gene recombinations in suspect lymphoproliferations: report of the BIOMED-2 Concerted Action BMH4-CT98-3936. Leukemia.

[4] Brüggemann M, White H, Gaulard P, Garcia-Sanz R, Gameiro P, Oeschger S, Jasani B, Ott M, Delsol G, Orfao A, Tiemann M, Herbst H, Langerak AW, Spaargaren M, Moreau E, Groenen PJTA, Sambade C, Foroni L, Carter GI, Hummel M, Bastard C, Davi F, Delfau-Larue MH, Kneba M, van Dongen JJM, Beldjord K, Molina TJ (2007). Powerful strategy for PCR-based clonality assessment in T-cell malignancies. Report of the BIOMED-2 Concerted Action BHM4-CT98-3936. Leukemia.

[5] Evans PAS, Pott C, Groenen PJTA, Salles G, Davi F, Berger F, Garcia JF, van Krieken JHJM, Pals S, Kluin P, Schuuring E, Spaargaren M, Boone E, González D, Martinez B, Villuendas R, Gameiro P, Diss TC, Mills K, Morgan GJ, Carter GI, Milner BJ, Pearson D, Hummel M, Jung W, Ott M, Canioni D, Beldjord K, Bastard C, Delfau-Larue MH, van Dongen JJM, Molina TJ, Cabeçadas J (2007). Significantly improved PCR-based clonality testing in B-cell malignancies by use of multiple immunoglobulin gene targets. Report of the BIOMED-2 Concerted Action BHM4- CT98-3936. Leukemia.

[6] Langerak AW, Molina TJ, Lavender FL, Pearson D, Flohr T, Sambade C, Schuuring E, al Saati T, van Dongen JJM, van Krieken JHJM (2007). PCR-based clonality testing in tissue samples with reactive lymphoproliferations: usefulness and pitfalls. A study from the BIOMED-2 Concerted Action BMH4-CT98- 3936. Leukemia.

[7] McClure RF, Kaur P, Pagel E (2006). Validation of immunoglobulin gene rearrangement detection by PCR using commercially available BIOMED-2 primers. Leukemia.

[8] Halldorsdottir AM, Zehnbauer BA, Burack WR (2007). Application of BIOMED-2 clonality assays to formalin-fixed paraffin embedded follicular lymphoma specimens: superior performance of the IGK assays compared to IGH for suboptimal specimens. Leuk Lymphoma.

[9] Liu H, Bench AJ, Bacon CM, Payne K, Huang Y, Scott MA, Erber WN, Grant JW, du MQ (2007). A practical strategy for the routine use of BIOMED-2 PCR assays for detection of B- and T-cell clonality in diagnostic haematopathology. Br J Haematol.

[10] Patel KP, Pan Q, Wang Y, Maitta RW, du J, Xue X, Lin J, Ratech H (2010). Comparison of BIOMED-2 versus laboratory-developed polymerase chain reaction assays for detecting T-cell receptor-gamma gene rearrangements. J Mol Diagn.

[11] Sandberg Y, van Gastel-Mol EJ, Verhaaf B, Lam KH, van Dongen JJ, Langerak AW (2005). BIOMED-2 multiplex immunoglobulin/T-cell receptor polymerase chain reaction protocols can reliably replace Southern blot analysis in routine clonality diagnostics. J Mol Diagn.

[12] Droese J, Langerak AW, Groenen PJ, Brüggemann M, Neumann P, Wolvers-Tettero IL, van Altena MC, Kneba M, van Dongen JJ (2004). Validation of BIOMED-2 multiplex PCR tubes for detection of TCRB gene rearrangements in T-cell malignancies. Leukemia.

[13] Langerak AW, Groenen PJ, Brüggemann M (2012). EuroClonality/BIOMED-2 guidelines for interpretation and reporting of Ig/TCR clonality testing in suspected lymphoproliferations. Leukemia.

[14] Cree IA, Deans Z, Ligtenberg MJ, for the European Society of Pathology Task Force on Quality Assurance in Molecular Pathology; Royal College of Pathologists (2014). Guidance for laboratories performing molecular pathology for cancer patients. J Clin Pathol.

[15] International Organization for Standardization (2012). ISO 15189:2012 Medical laboratories - particular requirements for quality and competence.

[16] Harris S, Bruggemann M, Groenen PJTA, Schuuring E, Langerak AW, Hodges E (2012). Clonality analysis in lymphoproliferative disease using the BIOMED-2 multiplex PCR protocols; experience from the EuroClonality group EQA scheme. J Hematop.

[17] Miller JE, Wilson SS, Jaye DL, Kronenberg M (1999). An automated semiquantitative B and T cell clonality assay. Mol Diagn.

[18] Greiner TC, Rubocki RJ (2002). Effectiveness of capillary electrophoresis using fluorescent-labeled primers in detecting T-cell receptor gamma gene rearrangements. J Mol Diagn.

[19] Kuo FC, Hall D, Longtine JA (2007). A novel method for interpretation of T-cell receptor gamma gene rearrangement assay by capillary gel electrophoresis based on normal distribution. J Mol Diagn.

[20] United Kingdom External Quality Assessment Services (2019) http://www.ukneqasli.co.uk/eqa-pt-programmes/molecular-genetics-programmes/igh-tcr-clonality-status-accredited. Accessed 22 October 2019

[21] College of American Pathologists (2019) https://documents.cap.org/documents/2019-surveys-catalog.pdf. Accessed 22 October 2019

[22] Wetenschappelijk Instituut Volksgezondheid/Institut scientifique de la Santé publique (2019) https://www.wiv-isp.be/QML/Informatiebrochure_EKE.pdf. Accessed 22 October 2019

[23] Qualitätssicherungs-Initiative Pathologie (2019) https://quip.eu/wp-content/uploads/2019/07/004_QuIP-Programm_25072019.pdf. Accessed 22 October 2019

[24] The Royal College of Pathologists of Australasia Quality Assurance Programs (2019) https://rcpaqap.com.au/products/molecular. Accessed 22 October 2019

[25] van Krieken JH, Normanno N, Blackhall F, Boone E, Botti G, Carneiro F, Celik I, Ciardiello F, Cree IA, Deans ZC, Edsjö A, Groenen PJTA, Kamarainen O, Kreipe HH, Ligtenberg MJL, Marchetti A, Murray S, Opdam FJM, Patterson SD, Patton S, Pinto C, Rouleau E, Schuuring E, Sterck S, Taron M, Tejpar S, Timens W, Thunnissen E, van de Ven PM, Siebers AG, Dequeker E (2013). Guideline on the requirements of external quality assessment programs in molecular pathology. Virchows Arch.

[26] Thunnissen E, Bovée JV, Bruinsma H (2011). EGFR and KRAS quality assurance schemes in pathology: generating normative data for molecular predictive marker analysis in targeted therapy. J Clin Pathol.

[27] Keppens C, Tack V, ’t Hart N (2018). A stitch in time saves nine: external quality assessment rounds demonstrate improved quality of biomarker analysis in lung cancer. Oncotarget.

[28] Keppens C, Dufraing K, Van Krieken HJ (2019). European follow-up of incorrect biomarker results for colorectal cancer demonstrates the importance of quality improvement projects. Virchows Arch.

[29] EuroClonality (2019) http://euroclonality.org. Accessed 22 October 2019

[30] International Organization for Standardization (2010). ISO 17043:2010 Conformity assessment - general requirements for proficiency testing.

[31] Website of the Biomedical Quality Assurance Research Unit, Clonality schemes (2019) http://kras.eqascheme.org/info/public/static/euroclonality.xhtml. Accessed 22 October 2019

[32] Hattie J, Timperley H (2007). The power of feedback. Rev Educ Res.

[33] Hicks PJ, Margolis MJ, Carraccio CL, Clauser BE, Donnelly H, Fromme HB, Gifford KA, Poynter SE, Schumacher DJ, Schwartz A, the PMAC Module 1 Study Group (2018). A novel workplace-based assessment for competency-based decisions and learner feedback. Med Teach.

[34] Flentje M, Böhmelt D, Sieg L, Eismann H (2019). Instructors for on-the-job training of advanced paramedics – definition of competencies and development of a quality management tool for a “High Responsibility Organization”. GMS J Med Educ.

[35] Dufraing K, Lierman E, Vankeerberghen A, Franke S, Dequeker E (2020). External quality assessment schemes for molecular diagnostic labs in Belgium - can we improve it?. Accred Qual Assur.

[36] Tembuyser L, Dequeker EM (2016). Endorsing good quality assurance practices in molecular pathology: risks and recommendations for diagnostic laboratories and external quality assessment providers. Virchows Arch.

