# Supplementary material for: Evaluation of a worldwide EQA scheme for complex clonality analysis of clinical lymphoproliferative cases demonstrates a learning effect
Source: Virchows Arch. 2021 Mar 8;479(2):365–76. doi: 10.1007/s00428-021-03046-0 (PMC8364525; doi:10.1007/s00428-021-03046-0)
Supplement: Supplementary file 1 — (PDF 227 kb). [file 428_2021_3046_MOESM1_ESM.pdf]

Title: Evaluation of a worldwide EQA scheme for complex clonality analysis of clinical lymphoproliferative cases demonstrates a learning effect.

Journal: Virchows Archiv

Authors: Cleo Keppens, Elke Boone, Paula Gameiro, Véronique Tack, Elisabeth Moreau, Elizabeth Hodges, Paul Evans, Monika Brüggemann, Ian Carter, Dido Lenze, Maria Eugenia Sarasquete, Markus Möbs, Hongxiang Liu, Elisabeth MC Dequeker, Patricia JTA Groenen

Correspondence: Dr. Patricia JTA Groenen
Radboud University Medical Center
Department of Pathology
Geert Grooteplein Zuid 10
6525 GA Nijmegen, the Netherlands
Email: Patricia.Groenen@radboudumc.nl

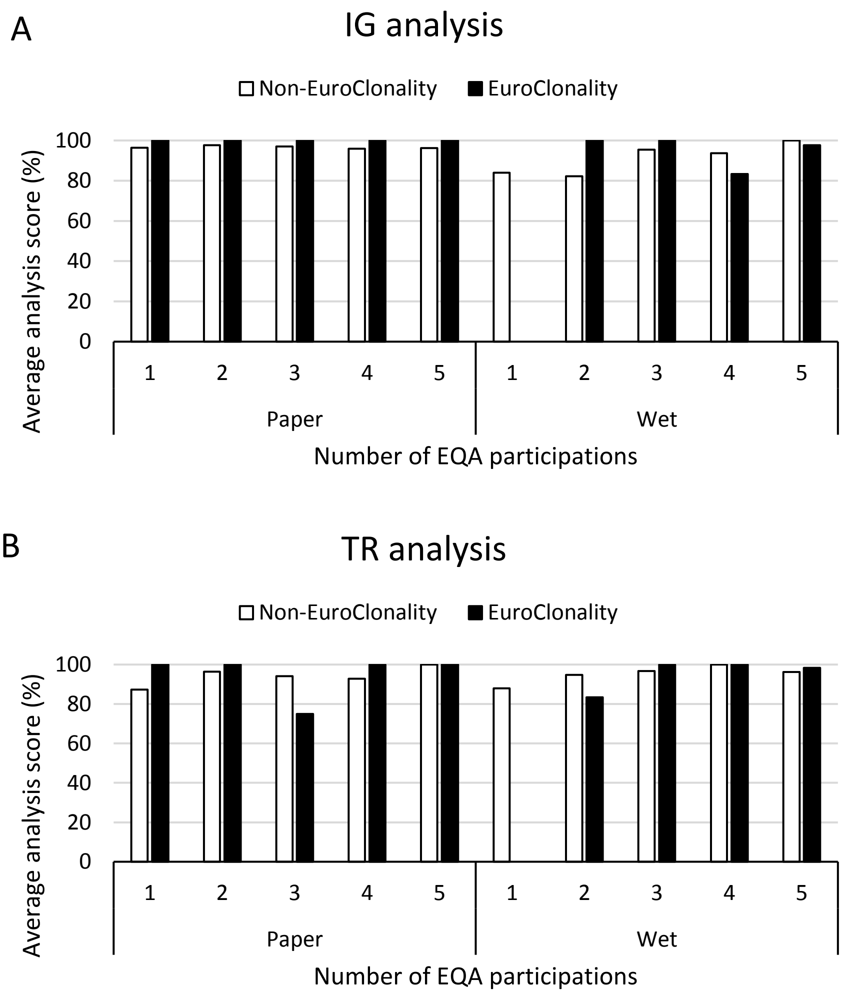

Supplemental Figure 1. Performance of EuroClonality and non-EuroClonality laboratories for paper and wet IG (A) and TR (B) cases.
Numbers represent the amount of EQA participations. All EuroClonality laboratories participated more than once in the wet cases and data is thus not available for EuroClonality labs who participated to only one scheme.
