# Supplementary material for: Evaluation of a worldwide EQA scheme for complex clonality analysis of clinical lymphoproliferative cases demonstrates a learning effect
Source: Virchows Arch. 2021 Mar 8;479(2):365–76. doi: 10.1007/s00428-021-03046-0 (PMC8364525; doi:10.1007/s00428-021-03046-0)
Supplement: Supplementary file 2 — (PDF 407 kb). [file 428_2021_3046_MOESM2_ESM.pdf]

Title Evaluation of a worldwide EQA scheme for complex clonality analysis of clinical lymphoproliferative cases demonstrates a learning effect.

Journal Virchows Archiv

Authors Cleo Keppens, Elke Boone, Paula Gameiro, Véronique Tack, Elisabeth Moreau, Elizabeth Hodges, Paul Evans, Monika Brüggemann, Ian Carter, Dido Lenze, Maria Eugenia Sarasquete, Markus Möbs, Hongxiang Liu, Elisabeth MC Dequeker, Patricia JTA Groenen

Correspondence Dr. Patricia JTA Groenen
Radboud University Medical Center
Department of Pathology
Geert Grooteplein Zuid 10
6525 GA Nijmegen, the Netherlands
Email: Patricia.Groenen@radboudumc.nl

Supplemental Table 1. (Inter)national providers of clonality testing EQA programs.

EQA provider	Region	Accredited provider?	# rounds per annum	Targets	Sample type	# samples distributed	criterium for successful performance	action in case of unsatisfactory performance	criteria for persistent poor performance	Action in case of unsatisfactory performance
BQA Research Unit KU Leuven in collaboration with EuroClonality Consortium [29,31]	Europe	yes, ISO17043: 2010[30]	1	IGH VH-JH, IGH DH-JH, IGK VK-JK, IGK V/intron-KDe, TRB VB-JB, TRB DB-JB, TRG VG-JG,	Paper based cases and extracted DNA from patient samples	10: 5 for IG and 5 for TR	maximum 1 error on total of 5 samples (for IG and TR separately)	Face-to-face discussion of EQA results during post-EQA workshop for EuroClonality Consortium affiliated labs	Max. 1 error on total of 10 samples over 2 rounds (for IG and TR separately)	No

Supplemental Table 1 *(Continued)*. (Inter)national providers of clonality testing EQA programs.

EQA provider	Region	Accredited provider?	# rounds per annum	Targets	Sample type	# samples distributed	criterium for successful performance	action in case of unsatisfactory performance	criteria for persistent poor performance	Action in case of unsatisfactory performance
UK NEQAS[20]	UK (European participants may also register)	yes, ISO17043: 2010 [30]	3	IGH VH-JH, IGH DH-JH, IGK VK-JK, IGK Kde, IGL, TCRB Vβ-Jβ, TCRB Dβ-Jβ, TCRG Vγ-Jγ, TCRD	Lyophilized cell-lines or patient derived material, 1 paper case	6: 1 sample for IG and 1 sample for TR each round, 1 paper case *per annum*	Incorrect outcome for 1 or 2 samples or no results submission	Communication to participants in report and performance letter, support and guidance (provision of repeat samples, telephone, email or face-to-face communications.)	2/3 unsuccessful trials	Communicated to Genetics NQAAP panel for UK laboratories only.
WIV/ISP (Sciensano) (via UKNEQAS)[20]	Belgium	yes, ISO17043: 2010[30]	See UK NEQAS							No
CAP[21]	USA	yes, CAP, CLIA	2	IGH, IGH/BCL2 major and minor, IGH/CCND1, IGK TRB, TRG	Extracted DNA sample	12: 3 samples per round, each sample in duplicate	at least 80% for a subspecialty or clerical errors or data omissions	laboratory to complete a Proficiency Testing Compliance Notice (PTCN) form, documenting corrective actions	3 consecutive unsuccessful trials or 3/4 or 2/2 trials	Cease patient/client testing for a period of 6 months. Perform 2 successful trials before reinstatements and submit documentation (investigation, corrective action, patient impact and retraining)

Supplemental Table 1 *(Continued)*. (Inter)national providers of clonality testing EQA programs.

EQA provider	Region	Accredited provider?	# rounds per annum	Targets	Sample type	# samples distributed	criterium for successful performance	action in case of unsatisfactory performance	criteria for persistent poor performance	Action in case of unsatisfactory performance
QuiP[23]	Germany	No (ongoing)	1	Immunoglobulin G heavy chains and T cell receptor gamma and T cell receptor beta	Extracted DNA sample	10: 5 for IG and 5 for TR	All cases must be identified correctly (100%) and if a participant has a technical issue with one sample they can order a new tube of that sample.	Legally QuIP cannot sanction poor performance. There are no official bodies to inform about persistent poor performers. Poor performers do not get a certificate but we always offer additional one-to one feedback and the possibility to talk about measures to improve performance.		
RCPAQAP[24]	Australasia	yes, ISO17043[30]	1	IgH,TCR, unspecified	Extracted DNA sample	5 (2019) 3 (2020)	assess each sample using the following criteria:<br><br>Concordant – Meets the expected response<br>Discordant – Deemed unacceptable or does meet the expected response<br>Not Assessed - The submission is unable to be assessed due to sample issues and/or assay sensitivity	No actions, but poor performers are reported to National Association of Testing Authorities, Australia (NATA), after which laboratories need to demonstrate improvement plans during accreditation audit		

Abbreviations: EQA, external quality assessment; CAP, College of American Pathologists; CLIA, Clinical Laboratory Improvement Amendments; IG, immunoglobulin gene; Quip, Qualitätssicherungs-Initiative Pathologie; RCPAQAP, The Royal College of Pathologists of Australasia Quality Assurance Programs; TR, T-cell receptor gene; UK NEQAS, United Kingdom External Quality Assessment Services; WIV/ISP, Wetenschappelijk Instituut Volksgezondheid/Institut scientifique de la Santé publique.
