# Supplementary material for: Evaluation of a worldwide EQA scheme for complex clonality analysis of clinical lymphoproliferative cases demonstrates a learning effect
Source: Virchows Arch. 2021 Mar 8;479(2):365–76. doi: 10.1007/s00428-021-03046-0 (PMC8364525; doi:10.1007/s00428-021-03046-0)
Supplement: Supplementary file 3 — (PDF 341 kb). [file 428_2021_3046_MOESM3_ESM.pdf]

Title	Evaluation of a worldwide EQA scheme for complex clonality analysis of clinical lymphoproliferative cases demonstrates a learning effect.
Journal	Virchows Archiv

Authors: Cleo Keppens, Elke Boone, Paula Gameiro, Véronique Tack, Elisabeth Moreau, Elizabeth Hodges, Paul Evans, Monika Brüggemann, Ian Carter, Dido Lenze, Maria Eugenia Sarasquete, Markus Möbs, Hongxiang Liu, Elisabeth MC Dequeker, Patricia JTA Groenen

Correspondence: Dr. Patricia JTA Groenen
Radboud University Medical Center
Department of Pathology
Geert Grooteplein Zuid 10
6525 GA Nijmegen, the Netherlands
Email: Patricia.Groenen@radboudumc.nl

Supplemental Table 2. Samples provided to participants per scheme year.

EQA year	EQA type	Subscheme	Origin	Final Clinical Diagnosis	Final Molecular interpretation	% Correct Results (# labs)
2014	Paper	IG	Bone marrow	Hairy Cell Leukemia	Clonality detected	98.4 (61/62)
				Plasmablastic lymphoma	Clonality detected	100.0 (62/62)
			Peripheral blood	Reactive lymphocytosis	Polyclonality detected	96.8 (60/62)
				Mature B-cell neoplasm	Clonality detected	100.0 (62/62)
			Skin tissue fresh	Primary cutaneous follicle centre lymphoma	Clonality detected	95.2 (59/62)
		TR	Bone marrow	Mycosis fungoides (with extra-cutaneous dissemination)	Clonality detected	96.7 (58/60)
			Peripheral blood	Sézary syndrome	Clonality detected	96.7 (58/60)
			Skin tissue fresh	Mycosis fungoides	Clonality detected	96.7 (58/60)
				Peripheral T-cell lymphoma, NOS	Polyclonality detected (no clonality detected)	96.7 (58/60)
				Mycosis fungoides	Clonality detected	100 (60/60)

Supplemental Table 2 *(Continued)*. Samples provided to participants per scheme year.

EQA year	EQA type	Subscheme	Origin	Final Clinical Diagnosis	Final Molecular interpretation	% Correct Results (# labs)
2015	Paper	IG	Spleen tissue FFPE	Diffuse large B-cell lymphoma, NOS	Clonality detected	100.0 (57/57)
			Stomach tissue fresh	Chronic gastritis	Oligoclonality/multiple clones detected or Clonality detected	91.2 (52/57)
		TR*	Lymph node FFPE	Peripheral T-cell lymphoma, NOS	Clonality detected	100.0 (56/56)
			Peripheral blood	Reactive lymphocytosis	Oligoclonality/multiple clones detected or Clonality detected	94.6 (53/56)
	Wet	IG	Lymph node fresh	Reactive lesion	Polyclonality detected	94.7 (54/57)
				Mature B-cell neoplasm	Clonality detected	94.7 (54/57)
			Lymph node FFPE	Follicular lymphoma (with t(14;18) detected by FISH)	Clonality detected	91.2 (52/57)
		TR	Lymph node fresh	Reactive lesion	Polyclonality detected (no clonality detected)	87.5 (49/56)
			Peripheral blood	T-cell large granular lymphocytic leukemia	Clonality detected	100.0 (56/56)
2016	Paper	IG	Lachrymal node FFPE	Reactive lesion	Polyclonality detected	96.3 (52/54)
			Bone marrow	Chronic Lymphocytic Leukemia	Clonality detected	100.0 (54/54)
			Skin tissue fresh	Extranodal marginal zone B-cell lymphoma	Clonality detected	100.0 (54/54)
		TR	Peripheral blood	Relapsed T-cell prolymphocytic leukemia	Oligoclonality/multiple clones detected	29.4 (15/51)
			Skin tissue fresh	Mycosis fungoides	Clonality detected	98.1 (50/51)

Supplemental Table 2 *(Continued)*. Samples provided to participants per scheme year.

EQA year	EQA type	Subscheme	Origin	Final Clinical Diagnosis	Final Molecular interpretation	% Correct Results (# labs)
2016	Wet	IG	Bone marrow	Multiple myeloma, relapse	Clonality detected	100.0 (54/54)
			Lymph node fresh	Follicular lymphoma (with t(14;18) detected by FISH)	Clonality detected	81.5 (44/54)
		TR	Lymph node FFPE	Reactive lesion	Polyclonality detected	92.2 (47/51)
			Peripheral blood	Relapsed T-cell acute lymphoblastic leukemia	Clonality detected	98.1 (50/51)
				Peripheral T-cell Non-Hodgkin Lymphoma, NOS	Clonality detected	100.0 (51/51)
2017	Paper	IG	Paravertebral mass FFPE	Diffuse large B-cell lymphoma, NOS	Clonality detected	100.0 (48/48)
			Bone marrow	Monoclonal B-cell lymphocytosis	Clonality detected	100.0 (48/48)
		TR	Bone marrow	Large granular lymphocyte leukemia	Clonality detected	100.0 (47/47)
			Skin tissue FFPE	Diagnosis not conclusive (early phase of parapsoriasis or mycosis fungoides could not be excluded) and further clinicopathological follow-up was advised	Polyclonality detected	100.0 (47/47)
			Skin tissue fresh	Mycosis fungoides	Clonality detected	80.9 (38/47)
	Wet	IG	Lymph node fresh	Follicular lymphoma (with t(14;18) detected by FISH)	Clonality detected	100.0 (48/48)
				Reactive lesion	Polyclonality detected	97.9 (47/48)
			Spleen tissue fresh	Nodal marginal zone lymphoma (with transformation to large B-cell lymphoma)	Clonality detected	100.0 (48/48)
		TR	Bone marrow	Reactive lesion	Clonality detected	100.0 (47/47)
			Lymph node fresh	Peripheral T-cell lymphoma, NOS	Clonality detected	97.9 (46/47)

Supplemental Table 2 *(Continued)*. Samples provided to participants per scheme year.

EQA year	EQA type	Subscheme	Origin	Final Clinical Diagnosis	Final Molecular interpretation	% Correct Results (# labs)
2018	Paper	IG	Spleen tissue FFPE	Diffuse large B-cell lymphoma, NOS	Clonality detected	100.0 (55/55)
			Tumour tissue FFPE	Plasma cell neoplasm	Clonality detected	92.7 (51/55)
			Umbilicus fresh	Diffuse large B-cell lymphoma, NOS	Clonality detected	100.0 (55/55)
		TR	Lymph node FFPE	T-cell Prolymphocytic Leukemia	Clonality detected	96.4 (53/55)
			Pleural fluid	Peripheral T-cell lymphoma, NOS	Clonality detected	100.0 (55/55)
	Wet	IG	Lymph node fresh	Extraosseous plasmacytoma	Clonality detected	100.0 (55/55)
			Thyroid gland isthmus FFPE	Reactive lesion	Polyclonality detected	94.5 (52/55)
		TR	Lymph node fresh	Peripheral T-cell lymphoma, NOS	Clonality detected	100.0 (55/55)
				Angioimmunoblastic T-cell lymphoma	Clonality detected	100.0 (55/55)
			Skin tissue FFPE	Reactive lesion	Polyclonality detected	90.9 (50/55)

*Data excludes one spleen tissue FFPE case (2015, paper TR case) with final clinical diagnosis of diffuse large B-cell lymphoma for which no consensus could be reached.
Abbreviations: DLBCL, diffuse large B-cell lymphoma; EQA, external quality assessment; FFPE, formalin-fixed paraffin embedded; FISH, fluorescence in-situ hybridization; IG, immunoglobulin gene; N/A, not applicable; NOS, not otherwise specified; TR, T-cell receptor gene.
