# Supplementary material for: Evaluation of a worldwide EQA scheme for complex clonality analysis of clinical lymphoproliferative cases demonstrates a learning effect
Source: Virchows Arch. 2021 Mar 8;479(2):365–76. doi: 10.1007/s00428-021-03046-0 (PMC8364525; doi:10.1007/s00428-021-03046-0)
Supplement: Supplementary file 4 — (PDF 345 kb). [file 428_2021_3046_MOESM4_ESM.pdf]

Title	Evaluation of a worldwide EQA scheme for complex clonality analysis of clinical lymphoproliferative cases demonstrates a learning effect.
Journal	Virchows Archiv
Authors	Cleo Keppens, Elke Boone, Paula Gameiro, Véronique Tack, Elisabeth Moreau, Elizabeth Hodges, Paul Evans, Monika Brüggemann, Ian Carter, Dido Lenze, Maria Eugenia Sarasquete, Markus Möbs, Hongxiang Liu, Elisabeth MC Dequeker, Patricia JTA Groenen
Correspondence	Dr. Patricia JTA Groenen<br>Radboud University Medical Center<br>Department of Pathology<br>Geert Grooteplein Zuid 10<br>6525 GA Nijmegen, the Netherlands<br>Email: Patricia.Groenen@radboudumc.nl

Supplemental Table 3. Overview of tubes and reagents used by the EQA participants.

Target	PCR test (described according to the EC assays)	All years		2018 (N=55 laboratories)						
		# tests (n=533)	% of tube included in analyses	# tests (n=110)	% of tube included in analyses	# laboratories who did not use the tube	# laboratories using non-EC LDTs	# laboratories using EC LDT (no commercial primer mixes)	# laboratories using commercialized EC tests (Invivoscribe)	# laboratories using Invivoscribe NGS assays
IG	IGH-VJ FR1 (tube A)	504	94.6	108	98.2	0	1	23	30	1
	IGH-VJ FR2 (tube B)	526	98.7	108	98.2	0	1	23	30	1
	IGH-VJ FR3 (tube C)	527	98.9	107	97.3	0	1	23*	30	1
	IGH-DJ (tube D)	242	45.4	49	44.5	28	0	14	13	0
	IGH-DJ (tube E)	146	27.4	26	23.6	39	0	6	10	0
	IGK-VJ (tube A)	476	89.3	100	90.9	4	0	25	25	1
	IGK-V/intron-Kde (tube B)	479	89.9	100	90.9	4	0	25	26	0
	IGL-VJ	111	20.8	24	21.8	39	0	9	7	0

Supplemental Table 3 *(Continued)*. Overview of tubes and reagents used by the EQA participants.

Target	PCR test (described according to the EC assays)	All years		2018 (N=55 laboratories)						
		# tests (n=524)	% of tube included in analyses	# tests (n=165)	% of tube included in analyses	# laboratories who did not use the tube	# laboratories using non-EC LDTs	# laboratories using EC LDT (no commercial primer mixes)	# laboratories using commercialized EC tests (Invivoscribe)	# laboratories using Invivoscribe NGS assays
TR	TRD	69	13.2	20	12.1	43	3	4	5	0
	TRG-VJ (tubes A and B)	511	97.5	159	96.4	1	2	22	29	1**
	Additional TRG (one tube) test used	/	/	9	5.5	0	1°	1°°	1°°°	0
	TRB-VJ (tubes A and B)	453	86.5	144	87.3	6	2	16	31	0
	TRB-DJ (tube C)	449	85.7	143	86.7	6	2	16	31	0

Tubes used for analysis were requested during all scheme years. Detailed information on the reagents used was only requested in 2018, which is why the information on additional TRG tubes used is not available for all scheme years. Analysis of IGL and TRD rearrangements is not necessary for all lymphomas, hence explaining the reduced number of tests. Commercialized EC tests (Invivoscribe) included the Invivoscribe IdentiClone Gene Clonality Assays, Invivoscribe Individual Master Mixes and Invivoscribe RUO IGH/IGK/IGL/TRCB/TCRG/TCRD Gene Clonality Assays. Invivoscribe NGS assays included the Invivoscribe LymphoTrack® Assays PGM.

*One participant used, in addition to the EC-LDT, a non-EC LDT primer mix for IGH-C FR3. **The Invivoscribe NGS assay is a TRG one tube test.

°One participant used an alternative TRG assay, non-EC LDT. °° One participant used the TRG one tube assay designed by EC [1], which interrogates the TRG rearrangements in one tube. °°°One participant used the Invivoscribe TRG 2.0 assay [1].

Abbreviations: #, number; EC, EuroClonality; IG, immunoglobulin gene; LDT, laboratory-developed test; N, number; NGS, next-generation sequencing; TR, T-cell receptor gene.

1. Armand M, Derrieux C, Beldjord K, Wabeke T, Lenze D, Boone E, Bruggemann M, Evans PAS, Gameiro P, Hummel M, Villarese P, Groenen PJTA, Langerak AW, Macintyre EA, Davi F (2019) A New and Simple TRG Multiplex PCR Assay for Assessment of T-cell Clonality: A Comparative Study from the EuroClonality Consortium. Hemasphere 3(3):e255. https:// doi.org/10.1097%2FHS9.0000000000000255.
